# The Role of Early Life Gut Mycobiome on Child Health

**DOI:** 10.1016/j.advnut.2024.100185

**Published:** 2024-02-03

**Authors:** Kayleigh Amber Rodriguez, Manoj Gurung, Rachelanne Talatala, Jolene R Rearick, Meghan L Ruebel, Kimberly E Stephens, Laxmi Yeruva

**Affiliations:** 1 Arkansas Children’s Research Institute, Little Rock, AR, United States; 2 University of Arkansas for Medical Sciences, Department of Pediatrics, Division of Infectious Diseases, Little Rock, AR, United States; 3 Microbiome and Metabolism Research Unit, United States Department of Agriculture, Agriculture Research Service, Little Rock, AR, United States; 4 Arkansas Children’s Nutrition Center, Little Rock, AR, United States

**Keywords:** mycobiome, mycobiota, microbiota, milk, gut, fungus, early life

## Abstract

The human gut microbiota is composed of bacteria (microbiota or microbiome), fungi (mycobiome), viruses, and archaea, but most of the research is primarily focused on the bacterial component of this ecosystem. Besides bacteria, fungi have been shown to play a role in host health and physiologic functions. However, studies on mycobiota composition during infancy, the factors that might shape infant gut mycobiota, and implications to child health and development are limited. In this review, we discuss the factors likely shaping gut mycobiota, interkingdom interactions, and associations with child health outcomes and highlight the gaps in our current knowledge of this ecosystem.

**Statement of Significance**

Gut mycobiota plays an essential role in early life leading to programming of child health and development. This review discusses the role of multiple factors on the infant gut mycobiota composition and function and its association with health outcomes. It also highlights the knowledge gap and proposes future directions.

## Introduction

The human gastrointestinal ecosystem, or microbiome (total bacterial genome in an environment), plays a significant role in shaping host physiology, immune function, metabolism, and the gut–brain axis [1,2]. Though bacteria, fungi, viruses, and archaea are present in the gastrointestinal ecosystem, most human microbiome research to date has focused primarily on the bacterial component. In addition, the first fecal microbiota (total bacterial community in an environment) transplant to treat *Clostridioides difficile* was carried out in the 1950s [3] and received renewed interest since the 1980s [[4], [5], [6]]. Microbiota research over the last several decades has led to approval of 2 fecal microbiota-based therapies for recurrent *C. difficile* infection by the United States Food and Drug Administration [7]. Although lower in diversity than bacteria, fungal colonization in the gastrointestinal system has experienced increased attention during the last decade. The composition and characteristics of mycobiota (total fungal communities in an environment) in healthy mammals and their interaction with other microbiota likely harbor important insights into child health and development. The commensal mycobiome provides a variety of essential roles in the metabolism and digestion of nutrients and provides antigens to shape the host-acquired immune system response [[8], [9], [10], [11], [12]]. Intestinal fungal changes are associated with disease in adults [13]; mucosal fungi actively modulate the innate and adaptive immune responses and have been associated with disease susceptibility (e.g., metabolic diseases, cancer, gastrointestinal disorders) [9,[14], [15], [16], [17], [18]]. Literature so far has shown the association of microbiota or mycobiota to host health and physiologic outcomes [[19], [20], [21], [22], [23], [24], [25], [26], [27], [28]]. However, we propose that the understanding of the whole ecosystem, including the mycobiota along with microbiota, will help develop future interventions to improve health. In this review, we aim to provide a current overview of the impact of early life factors on the gastrointestinal tract mycobiome (i.e., small and large intestine or fecal mycobiome) and its potential implications for host health and the gap in knowledge for future studies.

Despite evidence of early fungal colonization in the infant (i.e., 0–1 y) gastrointestinal tract, our knowledge of its effects on child (i.e., <14 y) health is limited. Recent evidence on microbiome composition and dynamics during early life development suggests that microbial effects on host immune function and metabolism can impact later health outcomes [[29], [30], [31]]. Our improved understanding of the development of the infant gastrointestinal tract mycobiome composition, factors involved in determining mycobiome composition, and mechanisms that underlie homeostasis will help formulate interventions for maintaining homeostasis and host health. Initially, we will discuss the diversity and composition of the mycobiome, factors shaping its development, and mechanisms by which it impacts host physiology. We will then review evidence on the impact of early life factors on the mycobiome and potential downstream risk of developing chronic diseases later in life. Finally, we will explore the potential therapeutic and/or nutritional interventions targeting the mycobiome during early life to promote lifelong health.

The scientific terminology for commensal bacteria and fungi is not consistent in the literature as “microbiome” is often used in reference to bacteria alone or collectively to refer to bacteria, fungi, viruses, and archaea. To avoid confusion, we use “microbiota” or “microbiome” to refer to bacteria and “mycobiota” or “mycobiome” (total fungal genomes in an environment) to refer specifically to fungi in the current review. For this review, we used the following search terms in the PubMed databases: “mycobiota and child health,” “mycobiota and infant,” “mycobiota and health outcomes or infant health outcomes,” and “mycobiome and child health.” The search terms “fungus” and “fungi” were excluded from this study due to the prevalence of literature on free living fungal organisms (such as mushrooms) unrelated to the human mycobiome. We also reviewed reference lists from PubMed related to gastrointestinal system mycobiome and child health to capture any additional literature missed with search terms to identify gaps in knowledge and suggest future studies to fill these limitations. This review provides a current synthesis on the role of early life factors in shaping the mycobiome and its implications for human health and highlights potential avenues for future research and therapeutic interventions.

## Diversity and Composition of the Mycobiome

As has been previously observed in human microbiota communities, the diversity and composition of the mycobiome are critical factors that influence other microbial gastrointestinal tract communities, host physiology, and host health outcomes. Also, the timeline, pattern of development, and mycobiome diversity in infants is similar to gut microbiome, and this could possibly influence the microbial community structure. It is important to note that variation in microbiome community structure exists in healthy populations [32]; however, certain alterations in mycobiome composition and diversity have been associated with the development of diseases [e.g., inflammatory bowel disease (IBD), asthma, obesity]. Increased diversity of the gut mycobiome is associated with a healthy immune system and reduced risk of chronic disease. Furthermore, gastrointestinal tract mycobiome diversity increases significantly throughout the first year of life [21,33]. This increased diversity likely confers protective effects during early development. For example, full-term infants with greater mycobiome diversity have reduced risk of developing allergies in later life [33]. Furthermore, preterm infants have decreased mycobiome diversity and experience increased risk of gastrointestinal comorbidities compared with full-term infants [34]. In addition, decreased diversity of the gut mycobiome, such as that seen with Crohn’s disease, is associated with increased inflammation and disease severity in adults [18]. However, it is currently unknown what portion of these gastrointestinal issues may be attributable to changes in the mycobiome compared with other causes due to lack of research on these topics.

## Early Life Factors Impact on Gastrointestinal Tract Mycobiota Colonization and Health Outcomes

**FIGURE 1:**
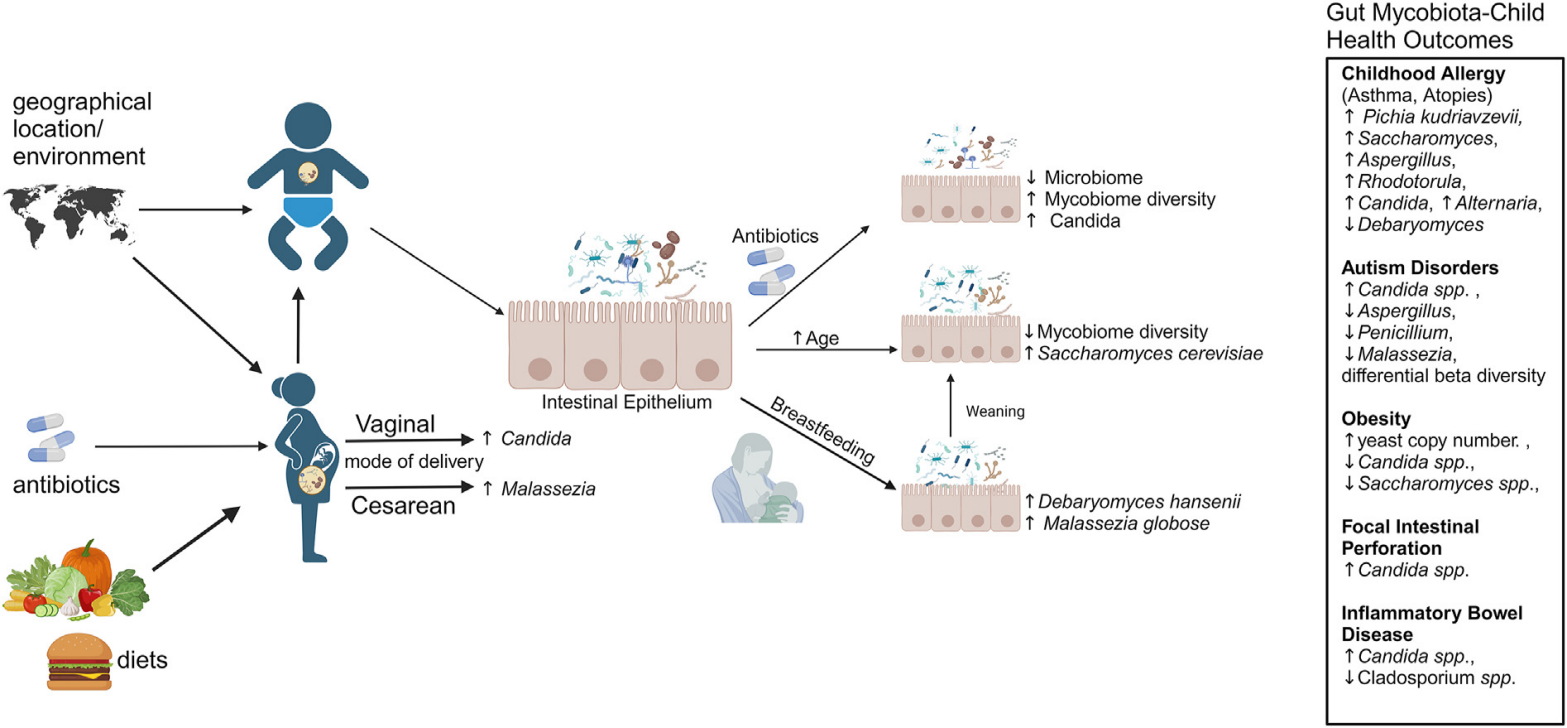
Gastrointestinal tract mycobiota impact on child health outcomes. The cartoon summarizes the current literature on various factors that influence gut mycobiota and, in turn, impact child health outcomes. Maternal factors such as geographic location, environment, diet, mode of delivery, and antibiotic use influence milk mycobiota composition. In addition, postnatal factors, including neonatal diet, antibiotic use, and age, influence infant gut mycobiota composition, function, and gut health. All these factors likely impact obesity, allergies, and chronic disease outcomes in children. Created with BioRender.com.

The development of gut mycobiota is affected by many factors, including mode of delivery [35], diets [36,37], environment [35,37,38], geographic location [35,38], and consumption of drugs or supplements [39,40]. Although colonization occurs at various body sites (e.g., mouth, skin, and gut) [41], we focus our discussion on the gastrointestinal tract mycobiota. We outline the factors shaping gastrointestinal tract mycobiota composition (Figure 1), the association of the mycobiota with health outcomes, mycobiota–microbiota interactions in the gastrointestinal tract, and resulting health outcomes in children.

### Maternal factors and birth mode

Mycobiomes are vertically transferred from mother to infant; an infant’s skin, oral, and anal mycobiomes are similar to their mother’s mycobiota regardless of the time point or body site sampled [41]. However, Amenyogbe et al. [42] provided evidence that the influence of maternal mycobiota on the colonization of infants may be limited, as 2 strains of *Candida glabrata* were shared between mother–infant pairs in low-resource settings during only the first month of life. Infants are exposed to mycobiota at birth through contact with the vaginal wall and skin [43] and skin-to-skin contact following delivery from mother to infant [44,45]. As expected, *Candida,* which is highly abundant on the vaginal walls, was the dominant fungal genus in the meconium of vaginally delivered infants, whereas *Malassezia* was predominantly found in infants delivered by Cesarean section [46]; a possible source of fungi is the skin of mother [47]. Existing studies report conflicting associations between the mode of delivery and composition of infant gastrointestinal tract mycobiota at 18 mo of age. In 1 study, *Trichosporon* was the most abundant in Cesarean-delivered infants [46]. However, these findings have not yet been replicated by other groups [45].

Several studies have investigated associations between mode of delivery, time since delivery, and milk mycobiota composition. In 1 study, *Saccharomyces cerevisiae* was the most abundant species followed by *Aspergillus glaucus* in transient milk (i.e., expressed on days 7–15 after birth) in Cesarean births but disappeared in mature milk (i.e., expressed on days 45–90) [48]. *Penicillium rubens* was the most abundant in mature milk samples [48]. In contrast, in mothers who experienced a vaginal delivery, *Malassezia globosa* was the most abundant fungus in both transient and mature milk samples [48]. In another study across countries, the relative abundance of *Cryptococcus*, *Candida smithsonii*, and *Ascomycota* species were higher in milk samples of females that delivered vaginally, and *Sistotrema*, *Malassezia restricta*, and *Davidiella tassiana* were abundant in samples delivered by Cesarean sections [35]. However, these findings should be considered cautiously, as the geographic locations of subjects differed and may better explain these differences in taxa [35]. Collectively, these studies highlight differences in fungal colonization in human milk through associations with mode of delivery and length of time since delivery. The mechanism of how the mode of delivery impacts mycobiota presence in human milk is unknown, and the functional relevance of these specific mycobiota to either mother or infant is also understudied.

Maternal diet, obesity, and probiotic supplementation impact the infant gastrointestinal tract microbiome [45,[49], [50], [51], [52], [53]]. Probiotics could restore the gastrointestinal tract microbiota to reduce inflammation and benefit the immune system and other physiologic functions in a host [54]. With the increase in popularity of use of probiotics, it would be important to understand the role of probiotic supplementation on mycobiota composition and function and determine how those changes impact the host. So far, only 1 study has investigated how supplementation of the maternal diet with a probiotic affects the gut mycobiome, suggesting a considerable knowledge gap in the field of mycobiota [45]. No significant effect of intake of maternal probiotic milk containing *Lactobacillus* and a *Bifidobacterium* species from 36 weeks of gestation until 3 mo after birth on infant gastrointestinal tract mycobiota composition was found. However, mothers who received supplementation compared with pregnant controls harbored a higher abundance of total fungal DNA and a lower abundance of *S. cerevisiae* in the maternal gut. Additional clinical studies with larger groups and the use of animal models are needed to understand how various maternal factors influence infants’ mycobiota composition and the synergistic effects of these factors on infants’ health outcomes.

### Geographic location and environment

Although mother–offspring transfer likely inoculates the infant gastrointestinal tract mycobiota in early life, other factors such as geography and housing environment are known to have a role in shaping its composition throughout development. The abundance of mycobiota present in the infant’s environment may also be an independent contributor to its colonization. Heisel et al. [44] hypothesized that environmental surfaces in the neonatal intensive care unit (NICU) and maternal milk impact the gastrointestinal tract mycobiota of neonates who spend the first months of their lives in intensive care units. Although NICU surfaces and human milk harbored different fungal communities, *Candida* and *Saccharomyces* species were prevalent in both. Nel Van et al. [55] and Amenyogbe et al. [42] analyzed the mycobiome diversity of low-resource rural African towns and found that *Ascomycota* and *Basidiomycota* are the 2 most abundant phyla in infant gastrointestinal tract mycobiota in South African children. However, *M. globosa* was prominent in infants living in rural communities in Ghana during the first 3 mo of life [42].

Not surprisingly, geographical location (i.e., Spain, Finland, South Africa, and China) is associated with differences in mycobiota composition of human milk, which is likely attributed to cultural factors and maternal diet intake (see preceding section) [35]. For example, *Malassezia* was lower and *Rhodotorula* and *Saccharomycetales* species were more abundant in South African breast milk samples than in samples from other locations (i.e., China, Spain, Finland). In samples from China, *Penicillium* and *Rhodotorula* were lower and *Malassezia* was highly abundant, whereas in Spanish and Finnish samples, *Saccharomyces* was higher. *Malassezia, Davidiella, Sistotrema*, and *Penicillium* genera were identified in milk samples collected from China, Spain, Finland, and South Africa, but their relative abundances varied [35]. *Saccharomyces* was the most abundant in Spanish and Finnish samples, whereas *Malassezia* was highly abundant in samples from China. In contrast, *Rhodotorula* and *Saccharomycetales* species were more abundant and *Malassezia* was less abundant than samples from China, Spain, and Finland [17]. These findings suggest that geographic location and environment contribute to the presence of these species in human milk. How these environmental factors influence mycobiota composition and diversity in later life remains to be explored. Although the gastrointestinal tract mycobiota community is dynamic over the first few years of life, insufficient evidence is available to determine the independent contribution of age in changing this community. Additional research would require larger cohorts in multiple geographic locations to determine the contribution of increasing age to mycobiota diversity in infants and children.

### Postnatal diet

Nutrition affects the gastrointestinal tract mycobiome [56], and postnatal diet is likely another influential factor in early life mycobiota composition [57]. Deficiency in micronutrients may also impact child health outcomes through gastrointestinal tract mycobiota, but more research is needed to determine the role and mechanisms of this relationship. Malnutrition is a global health issue that can increase risk of infection and gastrointestinal tract microbiota immaturity [[58], [59], [60], [61]]. International health programs include the use of micronutrient supplements (i.e., vitamins and minerals) as one of the ways to improve health outcomes in malnourished children. An improved understanding of the effects of these supplements on the gastrointestinal tract ecosystem would be beneficial to determine their association with health outcomes. Recently, children aged 12–24 mo were administered supplements containing vitamins and iron with or without zinc for 1 y. Subjects receiving supplements that lacked zinc showed increased *Mucormycetes* relative to the zinc-supplemented group [40]. *Mucormycetes* is a fungal taxon known to cause mucormycosis, a symptomatic respiratory and skin infection that can be fatal if left untreated. The addition of zinc reduced *Mucormycetes* amounts and may lower the ability of some eukaryotes to infect and persist in the host gut. In addition to the effects of zinc, vitamin A supplementation was associated with a higher fungal alpha diversity [55], suggesting the role of supplements in fungal community structure.

Recently, Gutierrez et al. [62] identified a positive association between *Saccharomyces* and *Malassezia* species and body mass index *z*-score between 1 and 5 y of age in children, suggesting an association for these fungal species with growth and development. Gut mycobiota of South African infants were colonized by *Ascomycota* and *Basidiomycota* [55], which were also abundant in the large intestine of piglets fed either human milk or formula milk from 2 to 21 d of life [57]. Piglets that were fed human milk from day 2 to day 21 had higher *M. globosa* in the cecal lumen at weaning compared with those fed formula milk, whereas the latter had *M. globosa* abundantly colonized in both the cecal lumen and distal colon at day 51 of life. *M. globosa* features prominently in the gut mycobiome during the first 3 mo of life of human milk-fed infants [42]. *Malassezia* species have the capacity to synthesize indoles to modulate the immune system [63]. The similarities in gut mycobiota composition between animal models and humans show promising translational applications for future mechanistic studies with animal models to understand the benefits of this species on host health. Altogether, this suggests that deficiencies in different micronutrients in infants and children may impact child health outcomes through gastrointestinal tract mycobiota, but more research is needed to determine the role and mechanisms of this relationship.

### Gastrointestinal tract mycobiota and health outcomes

In adults, mycobiota composition change has been reported in IBD [64], diabetes [65], schizophrenia [66], *C. difficile* [67], and Crohn’s disease [68]. Similar studies in children are limited compared to studies on gastrointestinal tract microbiota, and the majority focus on childhood allergies (e.g., asthma, atopy), which are common in children and can have long-term effects. Atopic diseases are inflammatory conditions characterized by IgE production in response to environmental allergens and include dermatitis, asthma, and rhinoconjunctivitis and are also associated with changes in gastrointestinal tract mycobiota [35,38]. Arrieta et al. [19] reported early life gastrointestinal system changes with yeast *Pichia kudriavzevii* and a higher mycobiota proportion than microbiota, though no significant differences were found in fungal alpha or beta diversity. Notably, fungal ecosystem alterations (*i.e.*, P. kudriavzevii, Saccharomyces) were more strongly associated with asthma risk than microbiota composition in this case–control study with control and atopic wheeze subjects [51]. Only 1 study reported fungal composition change in obese children with lower *Candida spp*. and *Saccharomyces spp*. than normal weight children [69], though childhood obesity is one of the leading health issues in children, suggesting a knowledge gap and need for future studies.

At least 2 studies reported an increased abundance of fecal *Rhodotorula* in children with atopies [21,70]. *Acremonium* and *Rhizopus* were the most abundant fungi in the feces of children who had outgrown atopic dermatitis within 1 y of age, whereas *Rhodotorula* was the most abundant in children whose symptoms persisted. These findings are in contrast to the no atopy healthy control children, in whom a higher abundance of *Kodamaea* and *Wickerhamomyces* was found [70]. Meta-proteomics revealed most of the fungal proteins of *Acremonium*, *Wickerhamomyces*, *Rhodotorula,* and *Rhizopus* were significantly increased in the persistent atopic dermatitis group, suggesting they were not only present but also metabolically active in infants’ guts. The *Rhodotorula* metabolites, RAN-binding protein 1 and glycerol kinase were hypothesized to be related to atopic dermatitis manifestation in these infants [70]. *Alternaria*, *Cladosporium*, *Saccharomyces*, *Acremonium*, and *Rhizopus* are also increased with atopies [21,69,71,72]. These limited studies provide some evidence that altered mycobiota composition contributes to atopic diseases during the first months of life through the production and release of metabolites that could provoke an altered inflammatory state, revealing a possible strategy to identify high-risk patients. The lack of existing studies limits our understanding of early life gut mycobiota and its impact on long-term health outcomes. Changes in *Candida* abundance are usually associated with health outcomes in later life. An increased *Candida* abundance is associated with atopies, focal intestinal perforation, autism disorder, asthma, and IBD, whereas decreased *Candida* is associated with type 1 diabetes and obesity [19,21,71]. Further studies are needed to determine if these associations are causal and/or disease-specific and present in early childhood as well as in adults (Table 1 [19,21,[69], [70], [71], [72], [73], [74]] and Figure 1).

**TABLE 1:** Mycobiota and health outcomes in children

Mycobiota taxa	Taxa	Health condition	Alpha and beta diversity	Sample size and age	Geographic location	Article (PMID)
*Saccharomyces, Aspergillus,* and *Pichia kudriavzevii*	Increased	AW	No difference between control and AW children	*n* = 27 (AW), *n* = 70 (healthy control) Ages: 3 mo to 5 y	Ecuador	Arrieta et al. [19] 2018 (29241587)
*Pichia kudriavzevii*	Increased	AW	—	*n* = 12 (AW), *n* = 115 (healthy control) Ages: 3 mo to 5 y	Canada	Boutin et al. [73] 2021 (33876729)
*Malassezia*	Decreased	Allergy	Change in beta diversity at 3-mo stool associated with sensitization to inhalant allergens at 5 y Increased alpha diversity at 3 mo of age and decreased alpha diversity at 1 y of age associated with inhalant atopy	*n* = 343 Ages: 3 mo, 1 y, and 5 y	Canada	Boutin et al. [21] 2021 (34060330)
*Rhodotorula* and *Candida*	Increased					
*Cladosporium*	Increased					
*Alternaria*	Increased					
*Saccharomyces*	Decreased					
*Candida*	Decreased					
*Rhodotorula*	Increased	Atopic dermatitis	Higher alpha diversity in group with persistent atopic dermatitis	*n* = 17 (Atopic dermatitis), *n* = 17 (healthy control) Ages: 9–12 mo	Thailand	Mok et al. [70] 2021 (34575786)
*Acremonium*	Increased					
*Rhizopus*	Increased					
*Candida*	Increased	FIP	—	*n* = 36 (FIP), Ages: 3–56 d	United States	Coates et al. [71] 2005 (15995004)
*Candida*	Increased	AD	No difference in alpha diversity	*n* = 40 (AD), *n* = 40 (neurotypical control) Ages: 3.6–17 y	Italy	Strati et al. [72] 2017 (28222761)
*Aspergillus*	Increased		Different beta diversity between control and AD subjects			
Malassezia	Increased					
Penicillium	Increased					
*Candida*	Increased	Obesity	—	*n* = 28 (obese), *n* = 33 (normal weight control) Ages: Average of 10.03 y	Italy	Borgo et al. [69] 2017 (27007700)
*Saccharomyces*						
*Candida parapsilosis* and *C. guilliermondii* (var. nitratophila)	Increased	IBD	Lower overall fungal diversity in IBD samples	*n* = 24 (IBD), *n* = 90 (healthy control) Ages: 4–20 y	United States	Chehoud et al. [75] 2015 (26083617)
*Cladosporium cladosporioides*	Decreased					
*Candida albicans*	Decreased	Type 1 diabetes T1DM	Higher fungal species diversity in T1DM subjects	*n* = 53 (T1DM), *n* = 30 (healthy control) Ages: Averages of 10.9 and 10.3 y	Poland	Kowalewska et al. [74] 2016 (27143864)

Abbreviations: AD, autism disorder; AW, atopic wheeze; FIP, focal intestinal perforation; IBD, inflammatory bowel disease; T1DM, type 1 diabetes mellitus.

— indicated no data were provided in the referenced article.

Similarly, another fungal species, *P. kudriavzevii,* was associated with later life asthma outcomes in Canadian and Ecuadorian children, and *Alternaria*, *Cladosporium*, *Saccharomyces*, *Acremonium*, and *Rhizopus* are also increased with atopies [21]. The most common fungal species that is associated with multiple health outcomes is *Candida* species. An abundance of *Candida* has been shown to increase in atopies, focal intestinal perforation, autism disorder, and IBD, whereas in type 1 diabetes and obesity, its abundance was lowered [19,[71], [72], [73], [74], [76]], suggesting fungal abundance change could be disease-specific (Table 1 and Figure 1). However, it is yet to be determined how early life mycobiota programs the gastrointestinal ecosystem and its impact on long-term health outcomes. From these studies, compositional changes in the mycobiome have been observed to associate with the development of atopic diseases that these relationships warrant further study for potential causative links.

## Mycobiota and Microbiota Interkingdom Communication

Despite the lack of literature describing the mycobiome, we would be remiss not to discuss the importance of how fungi interact with other microorganisms in the gastrointestinal tract. This ecosystem is characterized by a wide range of species, and the interaction between bacteria and fungi is specifically required for nutrient metabolism, modulation of the host immune system, and metabolite production [[77], [78], [79]]. Within the complex gastrointestinal ecosystem, bacteria and fungi compete for nutrients and influence activities in the host [[77], [78], [79]]. Further, these interactions may impact the overall health of the host and the state of the immune system. Our improved understanding of these complex interactions could provide valuable insights into how the microbiota and mycobiota shape our overall health and potentially develop strategies to maintain a beneficial gastrointestinal ecosystem.

The gastrointestinal tract mycobiome in healthy humans has low diversity and is dominated by few taxa compared with the microbiome. Germ-free mice (which are free of microorganisms) could provide a valuable model to identify a direct relationship between fungal colonization and the host immune function [70]. In the absence of gut microbiota, mycobiota grow in high abundance. Co-colonization with other microbiota alters the relative abundance of fungal species and significantly increases alpha diversity. Similarly, the presence of mycobiota changes the abundance and diversity of other members of the microbiota community. Mycobiota colonization promotes significant shifts in overall microbiota. For example, one species of fungus, *C. albicans,* is negatively associated with several bacterial species (e.g., *L. reuteri*, *Muribaculum intestinale*, *Flavonifractor plautii*, *Turicimonas muris*, *Bifidobacterium longum*, and *Clostridium clostridioforme*). However, it is positively correlated to other fungi: *I. orientalis*, *Candida parapsilosis*, *R. mucilaginosa*, and bacterial species *C. clostridioforme*, *Blautia coccoides, Enterococcus faecalis*, and *M. intestinale*. These shifts in microbial population were associated with decreased B cells, higher CD3^+^ T cells, and increased cytokines (i.e., IL-4, IL-6, IL-12, and IL-10) in both microbiota and mycobiota colonized mice when compared with microbiota or mycobiota colonization alone [80]. In addition, the interactions between both communities (mycobiota and microbiota) produce inflammation in the gastrointestinal tract. As an example, exclusive mycobiota colonization is insufficient to cause overt dextran sulfate sodium-induced colitis, whereas microbiota and mycobiota co-colonization increases colonic inflammation, which suggests that interkingdom interactions influence host physiologic responses.

Although antibiotics do not directly impact mycobiota, their use is associated with mycobiota overgrowth and alterations in community structure [74,81,82]. For example, infants treated with amoxicillin and macrolides for respiratory syncytial virus harbored greater mycobiota diversity and richness and a higher relative abundance of *Candida* compared with those who were not treated [39]. In addition, cephalosporin exposure increased invasive candidiasis in premature infants [83], and the pathogenic fungus *Trichosporon* is also highly abundant in the gut of children exposed to antibiotics during labor or delivery relative to antibiotic-unexposed children [46]. These changes in the composition of infant gut mycobiota following antibiotics likely reflect reduced competition/cooperation with resident gut microbiota, encouraging the growth of pathogenic/opportunistic species. To fully understand the role of mycobiota, we must examine the mycobiota both in isolation (as in germ-free mice) and in the presence of a complete gut ecosystem over time and space in both humans and animal models.

## Conclusion

With the advancement of methodologies like high-throughput sequencing, there is an increased interest in gastrointestinal tract mycobiota studies. However, most of the efforts have been on adults. In children, alterations in mycobiota have been associated with health conditions like allergies, type 1 diabetes, and autism symptoms. In addition, limited studies on mycobiota and child health outcomes, such as cognitive function, response to vaccinations, and gut health, resulted in a huge knowledge gap in this field. By 1 y of age, the fungal community of a child is largely established, and this may have beneficial effects against allergies and gastrointestinal disease later in life. Transmission of the mycobiome from mother to infant is a critical step in colonization altered by mode of delivery, feeding regime, and maternal diet. As the infant grows, mycobiota diversity is further impacted by housing environment, cultural practices, and geographic location, and it is unknown if these differences persist into later life. Large long-term studies among different cultures and geographic locations are needed to determine whether early mycobiota colonization directly determines later diversity and composition or if these factors are altered through later life experiences.

Postnatal diet composition influences the mycobiome, with known differences between formula- and breast-fed infants, as well as changes in children 1–2 y of age receiving vitamin supplementation. Although the mechanisms are poorly understood, alterations of mycobiota may contribute to inflammatory changes in early life that contribute to later disease development. It is currently unknown whether this change is causal, disease-specific, or immune-modulated. Studies of interactions between the fungal and other microbiome components have shown shifts in host immune cells and inflammation levels. The use of medications, such as antibiotics, that alter the host microbiome also alters the mycobiome community structure and likely contributes to fungal overgrowth or other diseases. With our current limited understanding of the role of fungal species in developing early microbiomes and the immune system, we must direct research toward maternal and infant hosts in various environmental conditions and across diverse geographic locations.

## Future Directions

1) Mycobiota are early colonizers of the infant gastrointestinal tract and contribute to the maintenance of gut homeostasis. As changes in fungal communities are associated with childhood asthma and atopies, most studies strive to identify associations between mycobiota composition and a health outcome and rarely report the full mycobiota profile. In addition, most studies in human subjects use only stool samples, as collecting samples from the intestine is challenging. Future studies on gastrointestinal tract mycobiota are needed, as stool mycobiota may not accurately represent the small and large intestine mycobiota, and the true core gut mycobiota remains to be fully defined in humans of all life stages.
2) A lack of mechanistic studies provides limited information on the impact of specific mycobiota species on host health and physiologic outcomes. Future studies are needed, such as germ-free mice or organoid models, that could recapitulate host physiology to fill the knowledge gap. In addition, creating an animal model with antifungal treatment would be useful to determine the role of gastrointestinal tract mycobiota on health outcomes. Determining important players in complex mycobiota–microbiota–host interactions is challenging yet essential to identifying causal mycobiota.
3) Systems biology approaches focusing on interactions between multiple systems, such as transkingdom network analysis [84], can provide insights into key mycobiota. The ecosystem in the gastrointestinal tract exists as a population of bacteria, fungi, viruses, and archaea. Future studies must be evaluated in longitudinal clinical cohorts and animal models to determine the competition/population dynamics and various factors impacting ecological niches.
4) In addition, longitudinal studies with metadata and mother–infant dyads are needed to understand the contribution of various maternal and postnatal factors and their synergy in mycobiota composition and function.
5) It is challenging to grow commensal fungi for particular species. It would be important to investigate cultural conditions and growth factors needed to advance the field of mycobiota. Leveraging various anaerobic culture techniques developed for microbiota research (i.e., Simulator of the Human Intestinal Microbial Ecosystem [85], Kobe University Human Intestinal Microbiota Model [86], and Gut-in-chip [87]) over the last decade will likely benefit the field of mycobiota.
6) Another potential area of research is to investigate the transmission of milk mycobiota from mother to infant either through internal (oro-entero-mammary) or external (retrograde inoculation) and whether mycobiota are present in the intramammary milk or only after breastfeeding via contact with maternal skin. The literature suggests the infant oral cavity is a potential source of milk mycobiota; conversely, maternal or environmental factors could be possible sources. The literature is conflicting in terms of specific species of mycobiota presence (i.e., *Malassezia*) with delivery mode and its presence in milk; thus, future studies are needed to clarify the source of the species in milk and infants. Another outstanding question is whether these mycobiota change the milk composition in-mammary gland and whether the gastrointestinal tract mycobiota uses the milk components (i.e., human milk oligosaccharides) like some well-known microbiota such as *Bifidobacterium.*
7) A high prevalence of mold and aflatoxins in low- and middle-income countries within food supplies [88,89] has been noted, and this may impact mycobiota composition and, in turn, child health [[90], [91], [92]]. It would be interesting to incorporate these variables in future studies.

## Terminology

Alpha diversity: Distribution of species abundance in a sample or community. Alpha diversity is determined by calculating richness (total number of species sample or community) and evenness (a measure of species abundance).

Beta diversity: Beta diversity measures the similarity or dissimilarity between 2 or more samples or communities.

Mycobiota: total fungal communities in an environment.

Mycobiome: total genomes of fungi in an environment.

Infant: 0–1 y of age.

Child: <14 y.

Gastrointestinal tract: small and large intestine regions.

## Author contributions

The authors’ responsibilities were as follows – LY and KE - conceptualized the topic and drafted the final manuscript; KR, RT, MG - conducted the literature review; MG - prepared the summary figure; MG, MR and JRR - edited and provided feedback.

### Conflict of interest

The authors report no conflicts of interest.

## Funding

The authors are supported by the Arkansas Children’s Research Institute (KES), the National Institutes of Health P20GM121293 (KES), the Horace C. Cabe Distinguished Chair in Infectious Disease (KES), and USDA-ARS Project #6026-51000-012-000D (MG, MLR, KES, RT, JRR, LY).
